# A Model for Urban Environment Instance Segmentation with Data Fusion

**DOI:** 10.3390/s23136141

**Published:** 2023-07-04

**Authors:** Kaiyue Du, Jin Meng, Xin Meng, Shifeng Wang, Jinhua Yang

**Affiliations:** 1 School of Optoelectronic Engineering, Changchun University of Science and Technology, Changchun 130022, China; 2 Zhongshan Institute of Changchun University of Science and Technology, Zhongshan 528400, China

**Keywords:** environment perception, instance segmentation, data fusion, Support Vector Machines, Markov Random Field, mean shift

## Abstract

Fine-grained urban environment instance segmentation is a fundamental and important task in the field of environment perception for autonomous vehicles. To address this goal, a model was designed with LiDAR pointcloud data and camera image data as the subject of study, and the reliability of the model was enhanced using dual fusion at the data level and feature level. By introducing the Markov Random Field algorithm, the Support Vector Machine classification results were optimized according to the spatial contextual linkage while providing the model with the prerequisite of the differentiation of similar but foreign objects, and the object classification and instance segmentation of 3D urban environments were completed by combining the Mean Shift. The dual fusion approach in this paper is a method for the deeper fusion of data from different sources, and the model, designed more accurately, describes the categories of items in the environment with a classification accuracy of 99.3%, and segments the different individuals into groups of the same kind of objects without instance labels. Moreover, our model does not have high computational resource and time cost requirements, and is a lightweight, efficient, and accurate instance segmentation model.

## 1. Introduction

The autonomous driving behavior of an unmanned vehicle is built on its perception of the surrounding environment for planning and decision making. Therefore, the strength of environment perception plays a key role in the intelligence and reliability of self-driving vehicles. Overall, environment perception can be subdivided into several tasks, such as road surface detection, dynamic/static object detection, and semantic segmentation, to name a few. Among them, semantic segmentation is the description of each point in the environment, which is a high-density representation task compared with other tasks. This representation is able to describe not only those objects in the environment that can generate motion trajectories, such as vehicles, pedestrians, etc., but also other steady-state objects, such as roads, buildings, etc.

To provide self-driving vehicles with a more fine-grained environment perception ability, instance segmentation is developed on the basis of semantic segmentation, which provides object categories along with labels of different objects in the same category. So, in an urban structured environment, instance segmentation maximizes the perception of the surroundings by self-driving vehicles.

Usually, the information acquisition of a vehicle’s surroundings is achieved with various sensors, such as LiDAR, cameras, radar, and acceleration sensors. Some scholars have developed semantic segmentation methods for outdoor environments relying on LiDAR pointcloud data alone, and provided theoretical support for the ability of a single sensor to accomplish the task of environment sensing. However, these methods require high-input pointcloud data, and the incomplete information degree and fewer features reduce perception accuracy [1,2]. There are also studies that chose the data fusion approach, because the advantage of fusion is that different sensors produce different forms of data for the same object, which allows the understanding of the environment to be improved in more dimensions. The semantic segmentation of the outdoor environment was completed by fusing LiDAR pointcloud data and camera image data, which enhanced environment segmentation accuracy [3,4,5,6].

Due to the popularity of deep learning in recent years, a large number of scholars have preferred to build network frameworks to explore pointcloud-based or image-based instance segmentation issues, because network frameworks are able to automatically learn complex data features and reduce the need for manual feature engineering [7,8]. However, deep learning is also known for its shortcomings, such as requiring a large amount of computational resources.

Thus, rather than using neural networks as in the above-mentioned research, it is more interesting to leverage the fusion of sensor data to make the segmentation of urban environment instances more reliable and resource-efficient. For this purpose, a lightweight and robust model consisting of Support Vector Machine (SVM), Markov Random Field (MRF), and Mean Shift was designed, hereafter called “SMS”. This model integrates classification, optimization, and segmentation tasks and does not require as much parameter tuning as the neural network algorithm, which has too much computational complexity, but also ensures excellent segmentation accuracy by introducing MRF to further optimize the results [7,9,10]. In general, MRF is used for more applications on images, such as image segmentation and image texture synthesis [11,12,13]. However, considering the Markov property, it is believed that it has some adaptability to optimization tasks based on spatial relations.

Therefore, our model, SMS, takes LiDAR pointcloud data and camera image data as inputs and completes the first fold fusion, i.e., data-level fusion, of pointcloud and image data using the projection relationship. In the process of adopting the SVM algorithm, the model discretizes the fusion data space with cubes, obtains the mapping relationship between pointcloud discrete blocks and image diffusion blocks according to the projection relationship, and stacks them after feature extraction, which completes the second fold fusion of pointcloud and image data, i.e., feature-level fusion. Then, the MRF algorithm is employed to optimize the classification results by leveraging the contextual linkage of spatially adjacent orthomosaics and to provide antecedent information for instance segmentation. Additionally, the Mean Shift algorithm completes instance segmentation among different individuals in the same category.

In summary, there are three main innovations:

1. In order to reduce the perceptual unreliability brought about by single-source data, the dual fusion of LiDAR pointcloud data and camera image data at the data level and feature level improves the perceptual strength of the model for the urban environment.

2. An MRF-based algorithm is utilized in our model to optimize the classification results by using the contextual linkage of spatially adjacent cubes to improve the model’s 3D instance segmentation of the urban environment.

3. This is a semi-labeled algorithm. Even though two kinds of data, LiDAR and camera data, are used, our annotation only requires the category annotation of the point cloud data, not detailed to individual annotation, and no annotation of the images.

The remainder of this paper is divided into four sections. Section 2 introduces the research background of instance segmentation, identifies gaps in contemporary research, and proposes a general process for solutions and contributions to the field and recent related technologies. Section 3 describes the methodology behind the instance segmentation model in this paper, and Section 4 further shows specific experiments using the model in this paper and presents a discussion of the experimental results. Finally, Section 5 summarizes the main work and the limitations of the proposed methodology and presents ideas for future work.

## 2. Related Work

In terms of object segmentation, approaches can be divided into those using whole segmentation and part segmentation [14,15]. In terms of data type, they can be divided into image instance segmentation, voxel instance segmentation, and pointcloud instance segmentation [16,17,18]. Due to the different data types, objects in images are usually continuous regions, while in 3D space, the discontinuity and the sparsity of the pointcloud increase the segmentation difficulty [19]. In terms of scene division, scenes can be divided into two types: indoor and outdoor environments. Compared with instance segmentation of an indoor environment, the difficulty with an outdoor environment is that it is easily affected by weather conditions, resulting in the absence of data, so more kinds of sensors are needed, but the enhanced segmentation accuracy also increases the difficulty of data processing [3,20,21].

One of the most common studies in outdoor environment segmentation is pointcloud semantic segmentation based on network design. For example, in order to reduce the need for large-scale training data and simplify the model, this approach converts pointcloud data into a bird’s eye view, and then uses migration learning for semantic segmentation [22]. Some semantic segmentation methods combine local and global contextual information, which better captures the shape and structure information of objects [23]. The following study implements real-time semantic segmentation by first converting continuous pointcloud data into a series of 2D depth maps and generating a corresponding binary mask at each time step. Then, these 2D depth maps and their corresponding binary masks are fed into the MOS network to obtain the final output: a binarized vector field containing information about the segmented regions of all moving objects [24]. However, these methods are semantic segmentation methods that use only a single source of data, i.e., LiDAR pointcloud data, and are highly dependent on pointcloud data, thus suffering from errors or missed detections when LiDAR is subject to certain limitations.

These next semantic segmentation methods are designed based on the fusion of pointcloud data and image data. After embedding the color information on the pointcloud, some studies generate segmentation results by fusing the fused pointcloud with the raw pointcloud features after lightweight convolutional neural network feature extraction, while others convert the pointcloud data into multiple 2D images and classify each image using a convolutional neural network, and finally fuse the classification results to obtain a label for each point in 3D space [5,6]. There are also studies that do not choose this data-level fusion approach, but complete the information exchange between images and pointcloud through a mutual learning strategy, and then complete the semantic segmentation in a migration-free learning process [7].

Instance segmentation can identify different individuals in the same category on the basis of semantic segmentation. Firstly, the instance segmentation results of these research teams are excellent, and both of them achieve point-level instance segmentation, but have different focuses. One of the teams is focused on segmenting moving objects, transforming pointcloud sequences into 4D voxels to extract motion features and using 4D sparse convolution to obtain motion features and inject them into the current scan. In the end, an upsampling fusion module is designed to output point-level labels. Another team introduces a dichotomous graph matching technique for end-to-end training, allowing the classifier to predict the labels of each instance independently and to adaptively learn the object morphology for a specific scene [10,25]. Another researcher draws attention to the pointcloud representation and designs a framework for polar coordinate top-view representation that can simultaneously learn semantic segmentation and category-independent instance clustering to solve the instance occlusion problem in urban street scenes. Of course, these instance segmentation methods are fine-grained, and at the same time have high demands in terms of computational resources and time cost, and have high hardware requirements when the scene size is large [26].

## 3. Methodology

**Figure 1:**
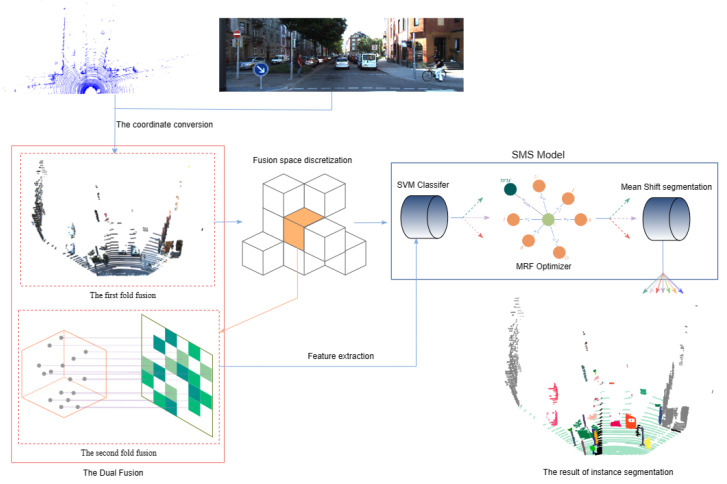
The overall framework of the instance segmentation proposed in this paper.

The flowchart of the instance segmentation algorithm of our paper is shown in Figure 1. It is a framework for the task of classifying, identifying, and segmenting the fusion data of LiDAR pointcloud data and camera image data.

### 3.1. Box Classification with SVM

In order to fuse heterogeneous data of the pointcloud data in the LiDAR coordinate system and the image data in the camera coordinate system, we employed the data-level fusion method, which projects the pointcloud on the image to obtain the fusion data through the coordinate conversion given in Formula (1) [27]:

(1) $$z_{L}\left[\begin{array}{c} u\\ v\\ 1 \end{array}\right]=P\times R\times T_{\mathrm{velo}\;}^{\mathrm{cam}\;}\times \left[\begin{array}{c} x_{L}\\ y_{L}\\ z_{L}\\ 1 \end{array}\right]$$

where *P* is the camera’s internal reference matrix, size 3×4, *R* is the correction rotation matrix, size 4×4, and $T_{velo}^{cam}$ is the conversion matrix from the LiDAR coordinate system to the camera coordinate system, containing the rotation matrix and translation vector, size 4×4. Additionally, $\left(x_{L},y_{L},z_{L}\right)$ is the pointcloud coordinates under the LiDAR coordinate system, and $\left(u,v\right)$ is the coordinates of the pixel under the image coordinate system after projection.

**Figure 2:**
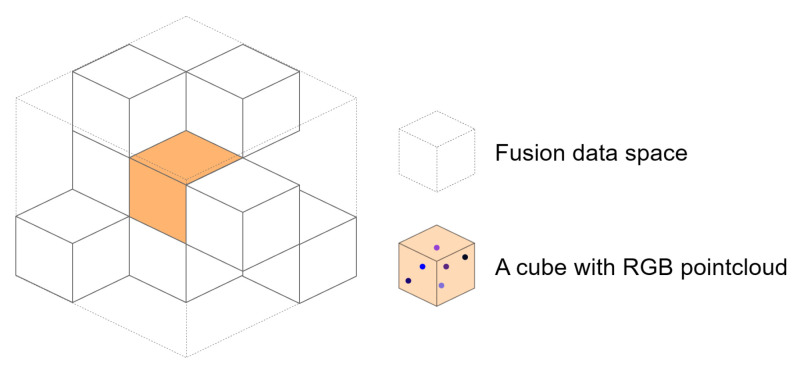
Fusion data space segmentation schematic.

We discretized the fusion data space to generate a large set of closely spaced cubes with the specific edge length. The cubes are divided into two states, occupied and non-occupied, and the occupied state contains a varying number of pointcloud data points with RGB information, as shown in Figure 2. Treating every cube as the processing object reduces the high computational consumption brought about by using each pointcloud as the processing object, while not losing the information brought about by using the voxel as the processing object.

For the feature extraction of pointcloud data within the cube, the features used contain the average reflectance, the average height, the height difference, the eigenvalues and eigenvectors of the covariance matrix of 3D coordinates, and the local spatial association feature. Reflectivity represents the data obtained by LiDAR during scanning, which reflects the material properties of the object’s surface. The height and the height difference reflect the undulating state of the object within the cube. The eigenvalues and eigenvectors of the covariance matrix in 3D coordinates reflect the shape characteristics of the object, such as whether it is large and flat, pole-like, or scattered [28,29].

**Figure 3:**
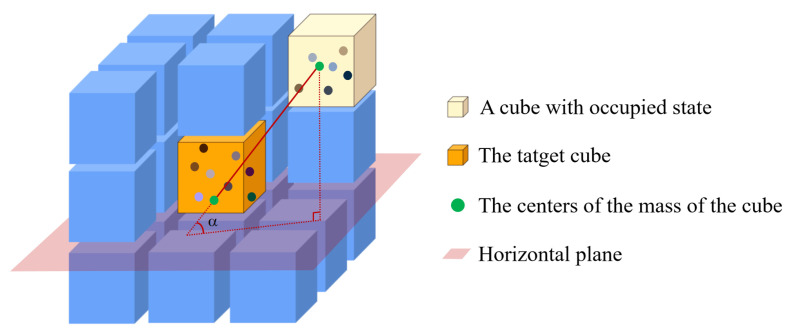
Illustration of the local spatial association feature of the target cube and another occupied cube.

The local spatial association feature is a feature designed to denote the pointcloud characteristics of a cube. We assume that a target cube in an occupied state in the fusion space will be wrapped by 26 other surrounding cubes, and the tangent values of the angle between the line that connects the center of mass coordinates of two cubes with the horizontal plane when the enclosing cubes are in the same occupied state constitute the local spatial association feature. The principle is shown in Figure 3.

**Figure 4:**
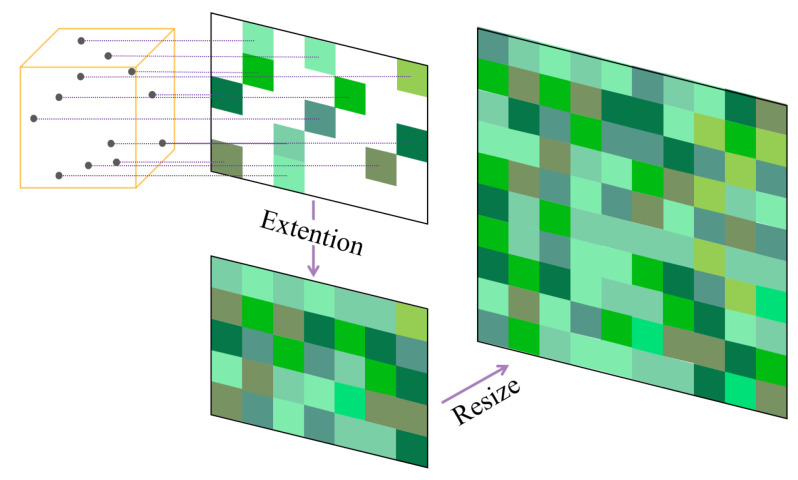
Illustration of local spatial association features of the target cube and another occupied cube.

Feature extraction was performed after structuring the discrete pixel points within the cube, and the extracted features contained color moments, the discrete Fourier transform, the grayscale covariance matrix, and local half-variance texture features. The principle of discrete pixel structuralization processing is shown in Figure 4.

Color moments are a representation of color features that describe the surface properties of an image region corresponding to an object [30]. Texture features exhibit the regular characteristics of pixel distribution and arrangement in an image, which are usually obtained by statistical means, such as the Fourier transform of image regions and grayscale covariance matrix [31,32,33]. As a kind of statistic to describe the spatial variability of random variables, semi-variance depends on the distance and direction of discrete points and reflects the autocorrelation between points, which provides an unbiased description of the spatial variation scale and pattern of image regions by describing the instability of image regions. The local semi-variance texture feature is designed according to this feature of semi-variance.

The local semi-variance texture feature is a feature designed to represent the characteristics of a pixel block in a cube based on the distribution and arrangement of pixels. The calculation rules of the local semi-variance texture feature are as follows: three directions of 0°, 45°, and 90° are set, and nine distances are set in each direction. The combination of half of the sum of the squares of the differences of the grayscale values at the ends of all the distances in all directions together constitute the local semi-variance texture feature. The mathematical expression is given in Formula (2).

(2) $$semi\_V_{direction}^{step}=\frac{1}{2N_{step}}\sum_{i=1}^{N_{step}}{\left[p\left(x\right)-p\left(x+step\right)\right]}^{2}$$

where, $semi\_V_{direction}^{step}$ means the semi-variance texture feature in some direction (direction = 1–3) with some step (step = 1–9), $N_{step}$ denotes the number of all pairs of pixels with distance step, $p\left(x\right)$ is the current point’s pixel value and $p\left(x+step\right)$ is the pixel value at the point separated by the step from the current point.

**Figure 5:**
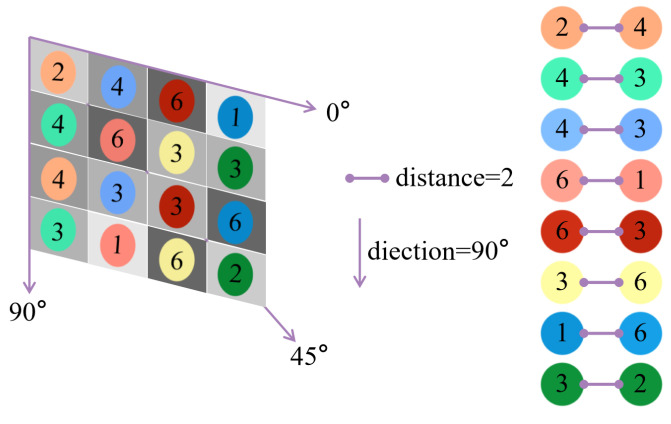
Illustration of the local spatial association feature of the target cube and another occupied cube.

Figure 5 shows a simplified schematic diagram of one of the procedures to calculate local semi-variance texture features, and the numbers represent the grayscale value of each pixel. The directions are 0°, 45°, and 90°, and the distances are 1, 2, and 3. From the figure, it is clear that when the direction is taken as 90° and the distance is taken as 2, there are 8 pairs of pixels. At this time, according to Formula (2) there is:

(3) $$\begin{array}{c} semi\_V_{90^{\circ }}^{2}=\frac{1}{2\times 8}\times \left[{\left(2-4\right)}^{2}+{\left(4-3\right)}^{2}+{\left(4-3\right)}^{2}+{\left(6-1\right)}^{2}\right.\\ \left.+{\left(6-3\right)}^{2}+{\left(3-6\right)}^{2}+{\left(2-6\right)}^{2}+{\left(3-2\right)}^{2}\right] \end{array}$$

Similarly, when the combination of direction and distance is taken, the local semi-variance texture feature of the simplified schematic is:

(4) $$\begin{array}{c} semi\_V=\left[semi\_V_{0^{\circ }}^{1},semi\_V_{0^{\circ }}^{2},semi\_V_{0^{\circ }}^{3},semi\_V_{45^{\circ }}^{1},semi\_V_{45^{\circ }}^{2},\right.\\ \left.semi\_V_{45^{\circ }}^{3},semi\_V_{90^{\circ }}^{1},semi\_V_{90^{\circ }}^{2},semi\_V_{90^{\circ }}^{3}\right] \end{array}$$

The features extracted from the pointcloud and the pixel block form the cube feature matrix with the manner of the cascade. We iterate through all the cubes to obtain the fusion feature matrix of the current frame of pointcloud data and the corresponding image.

### 3.2. Classification Refined with the MRF

**Figure 6:**
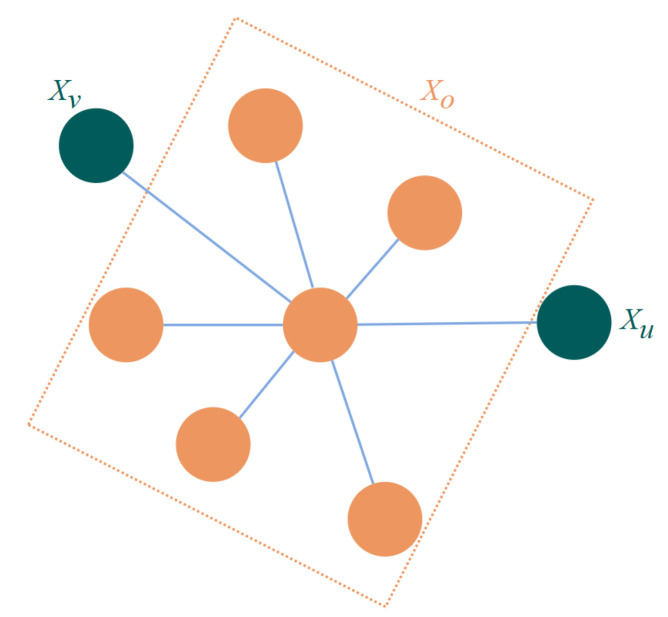
Undirected graph illustrating the conditional independence.

A Markov Random Field (MRF) is a set of random variables with Markov properties, described by the undirected graphical model in the probabilistic graphical model, i.e.,:

(5) G=(V,E)

where *G* is an undirected graph, *V* denotes the combination of nodes, and *E* denotes the combination of edges. Usually, nodes represent a random variable or a set of random variables, and edges represent the relationship between two connected nodes. As shown in Figure 6, an undirected graph, for any two nodes in the graph that are not connected by edges, such as $X_{u}$ and $X_{v}$, node $X_{u}$ and node $X_{v}$ are conditionally independent given the remaining node $X_{o}$, so this is a Markov Random Field.

**Figure 7:**
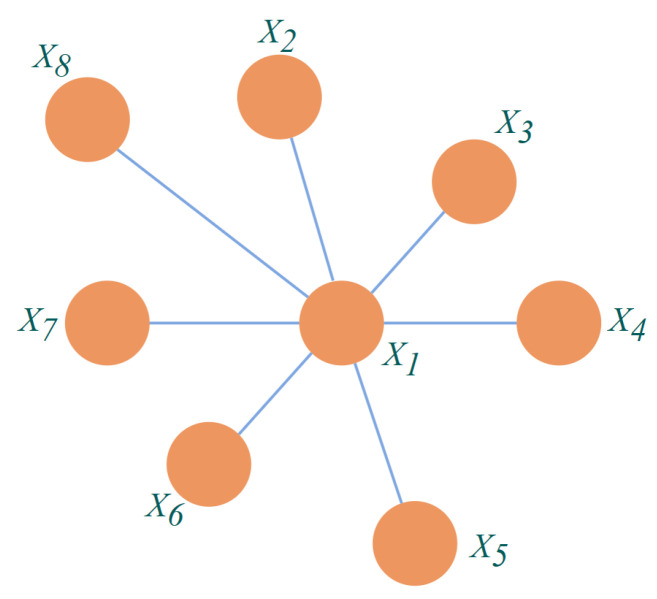
An undirected graph with 8 nodes.

For a subset of nodes in an undirected graph, if any two nodes in the subset are connected by edges, the subset of nodes is called a “clique”. When adding another node to a clique, it is impossible to form a clique, and the clique is called a “maximal cluster”. As in Figure 7, in this Markov Random Field, $\left\{X_{1},X_{2}\right\}$, $\left\{X_{1},X_{3}\right\}$, $\left\{X_{1},X_{4}\right\}$, $\left\{X_{1},X_{5}\right\}$, $\left\{X_{1},X_{6}\right\}$, $\left\{X_{1},X_{7}\right\}$, and $\left\{X_{1},X_{8}\right\}$ are all the cliques of the undirected graph and also the maximal cliques of the undirected graph [34].

The joint probability distribution of Markov Random Fields is defined by the potential function of a maximal clique. As an example, in Figure 7, the variable $X=\{X_{1},X_{2},\ldots ,X_{8}\}$, the set consisting of all maximal cliques *Q* is *C*, Q∈C, the subset of variables corresponding to *Q* is $X_{Q}$, and the potential function on Q is $\varphi_{Q}$; then, the joint probability is:

(6) $$P\left(X\right)=\frac{1}{Z}\prod_{Q\in C}\psi_{Q}\left(X_{Q}\right)$$

In which $Z=\sum_{X}\prod_{Q\in C}^{}\psi_{Q}\left(X_{Q}\right)$ is the normalization factor, which is a constant and generally does not need to be calculated. To ensure the non-negativity of the potential functions, it is usual to let:

(7) $$\psi_{Q}\left(X_{Q}\right)=e^{-H_{Q}\left(X_{Q}\right)}$$

Thus, the joint probability distribution of Formula (6) is expressed as

(8) $$P\left(X\right)=\frac{1}{Z}\prod_{Q\in C}e^{-H_{Q}\left(X_{Q}\right)}=\frac{1}{Z}\cdot e^{-\sum_{Q\in C}^{}H_{Q}\left(X_{Q}\right)}$$

Let:

(9) $$E\left(X\right)=\sum_{Q\in C}^{}H_{Q}\left(X_{Q}\right)$$

Furthermore, we refer to $E\left(X\right)$ as the energy function. Therefore, to maximize the joint probability, the energy function needs to be minimized.

**Figure 8:**
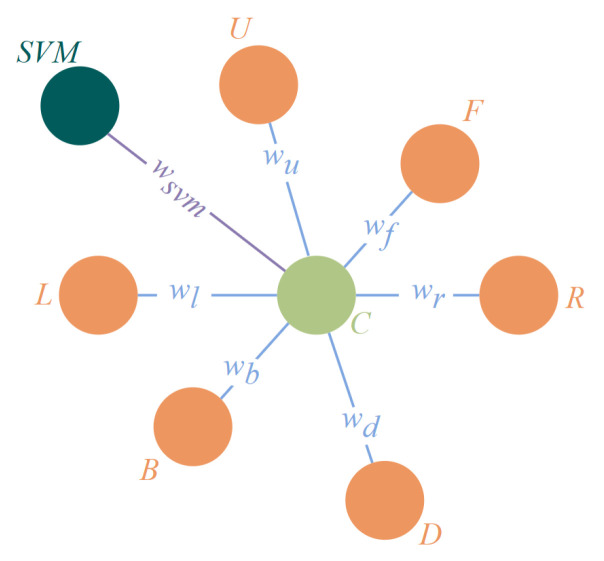
The MRF model designed in our algorithm.

Taking advantage of the ability of the neighborhood system to analyze spatial relationships, the Markov Random Field is applied to our algorithm. It is assumed that the value of a location in the random field is only related to the values of the locations adjacent to it, and not to the values of other non-adjacent locations. In our algorithm, the category of a cube itself is considered to be related only to the classification result of the SVM for it and the category of the cube adjacent to it. This is used as the basis for building the MRF model, as follows. With this as a basis, the MRF model of the paper is built, as shown in Figure 8. It is considered that the correction of node *C* is influenced by two parts: one is the nodes adjacent to it in terms of spatial position, and the other is the classification status of the SVM compared with the node to be corrected in the previous subsection.

The first part of the nodes constitutes the set of spatial position nodes: space_position$\_node\_set=\left\{U,D,L,R,F,B\right\}$. The potential functions of the maximal cliques formed by these nodes and the node to be corrected are designated as:

(10) $$\varphi_{position}=e^{-\left(position-C\right)},\;position\in \left\{U,D,L,R,F,B\right\}$$

The second part of the potential function is designed as:

(11) $$\chi_{svm}=e^{-\left(SVM-C\right)}$$

To further convey the influence of these nodes on the node to be corrected, however, rules for the values of the edges are defined:

(12) $$W_{node}=\left\{\begin{array}{c} 1-\frac{1}{N+1}\\ 0\\ svm\_accruracy \end{array}\right.$$

where *N* denotes the total number of occupied state nodes in the set of spatial position nodes. When the number of occupied state nodes in the spatial_position_node_set is higher, the nodes in the spatial_position_node_set have a greater influence on the correction-pending node. When a node in the spatial_position_node_set is a non-occupied state, it is logical that this node cannot have a correction capability for the node to be corrected. svm_accuracy is an explicit value of the classification accuracy of the SVM algorithm in the previous subsection.

The joint probability distribution of the MRF model in this paper is obtained by combining Formulas (10)–(12):

(13) $$P=W_{node}\cdot \chi_{svm}+W_{node}\cdot \varphi_{position}$$

### 3.3. Instance Segmentation with Mean Shift

The Mean Shift algorithm is a density-based non-parametric clustering algorithm that identifies the cluster centroids without specifying the number of clusters in advance. The core idea is that all points converge to a peak along the direction of gradient ascent, and the points that converge to the same peak belong to a cluster.

For a given *d*-dimensional space of *n* samples $x_{i}$, i=1,2,3,…,n, the Mean Shift vector for any point *X* in the sample space is represented as:

(14) $$M_{h}\left(X\right)=\frac{1}{k}\sum_{X_{i}\in S_{h}}^{}\left(X_{i}-X\right)$$

Among them, *k* denotes the number of points in the sample whose distance to $x_{i}$ is less than the spherical radius *h*, and $s_{h}$ denotes the high-dimensional spherical region of radius *h*, which is defined as:

(15) $$S_{h}\left(X\right)=\left(y|\left(y-x\right){\left(y-x\right)}^{T}\leq h^{2}\right)$$

This is the original vector form of the Mean Shift algorithm, which is simply a gradient-based ascending process and has the same contribution value for each point in the $s_{h}$ region [35]. However, in reality, the contribution value of each point in the $s_{h}$ region is related to the distance. To solve this problem, an improved Mean Shift algorithm was later developed by adding a kernel function that emphasizes the variable contribution values of each point in the $s_{h}$ region, resulting in an improved version of the vector form [36]:

(16) $$M_{h}\left(X\right)=\frac{\sum_{X_{i}\in S_{h}}^{}\left[K\left(\frac{X_{i}-X}{h}\right)\left(X_{i}-X\right)\right]}{\sum_{X_{i}\in S_{h}}^{}\left[K\left(\frac{X_{i}-X}{h}\right)\right]}$$

where $K\left(\frac{X_{i}-X}{h}\right)$ is a Gaussian kernel function with a functional expression of:

(17) $$K\left(X_{1},X_{2}\right)=K\left(\frac{X_{1}-X_{2}}{h}\right)=\frac{1}{\sqrt{2\pi }h}e^{-\frac{{\left(X_{1}-X_{2}\right)}^{2}}{2h^{2}}}$$

where *h* is the bandwidth, which is the radius of the high-dimensional sphere $s_{h}$.

After the previous two subsections, the classification of all discrete cubes in the fusion data space is already known, with the current classification distribution also being scattered. It is known that a particular cube belongs to a category, but there is no idea which cubes are one object. Thus, the Mean Shift is applied to our algorithm to distinguish the same type of foreign objects.

For a particular class in the category space, it is not obvious how many objects are contained in the class, but it is obvious that $s_{h}$ is a three-dimensional space of the sphere domain. The coordinates of the three-dimensional center of mass of all cubes under that class have been obtained. According to Formula (16), the coordinates of the position with the highest density in the current sphere space are obtained, and this is used as the new three-dimensional sphere center, and we continue on to calculate the position with the highest density in the new sphere. We iterate this process until the distance between the center of a particular sphere and the position of the highest density is extremely narrow, at which point the cube in which all the coordinates of the sphere are located is one object in the current class.

**Figure 9:**
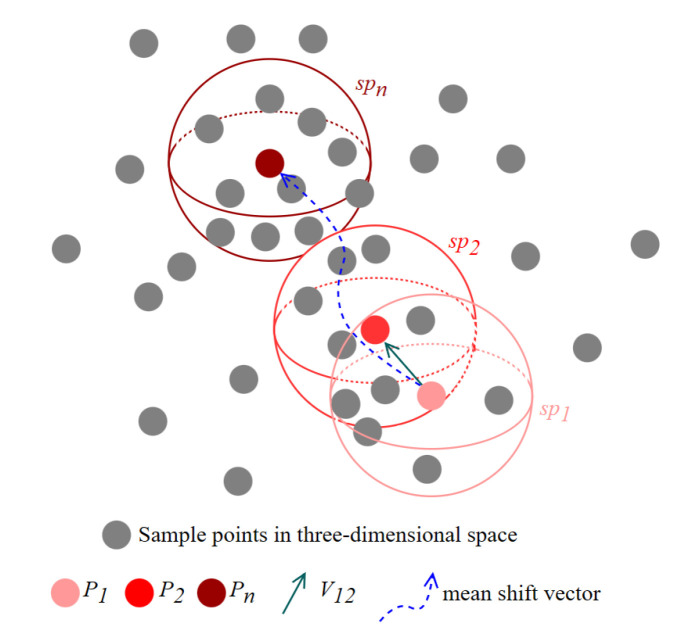
The principle diagram of the Mean Shift algorithm to find the cluster center.

The following Figure 9 illustrates the schematic diagram of the Mean Shift algorithm. Using gray dots to represent samples in three-dimensional space, $P_{1}$ is any one of them. A spherical domain $SP_{1}$ with $P_{1}$ as the center of the sphere contains several samples, and the mean shift vector $V_{12}$ is obtained according to Formula (16), which leads the center of the sphere $P_{1}$ to a location with higher density, that is, the endpoint $P_{2}$ of the vector $V_{12}$, with $P_{2}$ as the center of the new spherical domain $SP_{2}$. The mean shift vector is calculated again and so on until the highest density of the center of the sphere $P_{n}$ is available, and the samples in the spherical domain $SP_{n}$ are regarded as a cluster.

## 4. Experiment and Results

**Figure 10:**
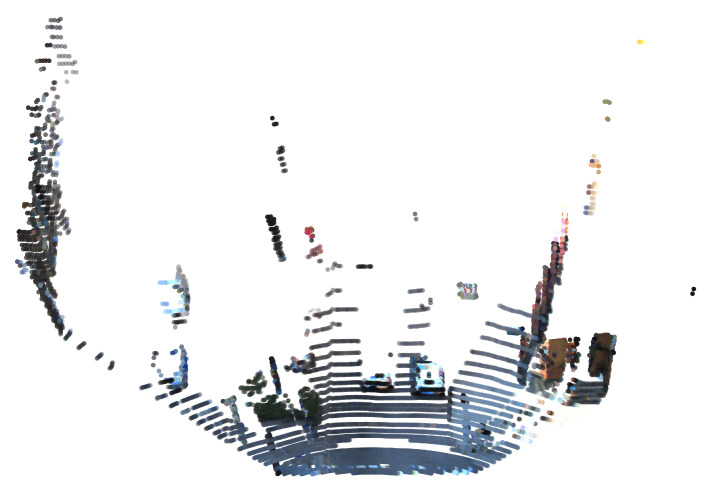
The pointcloud with RGB in the fusion space.

The dataset used in our experiments is the KITTI dataset. After the conversion according to the coordinate conversion formula between the LiDAR pointcloud and the camera image on the KITTI official website, a new 3D fusion space is obtained, as shown in Figure 10. The color information is attached to each pointcloud in the space, and the first fold data fusion in this paper is completed at this time. Through several experiments, it was decided to use a cube with an edge length of 40 cm to discretize the fusion space, since the cube at this value will not affect the accuracy of segmentation of the environment. The discrete operation generates a number of cubes with occupied states, and taking each cube as the feature extraction target reduces the computational consumption by more than using points as the processing object, and reduces the data loss phenomenon by more than using voxels as the processing object.

**Figure 11:**
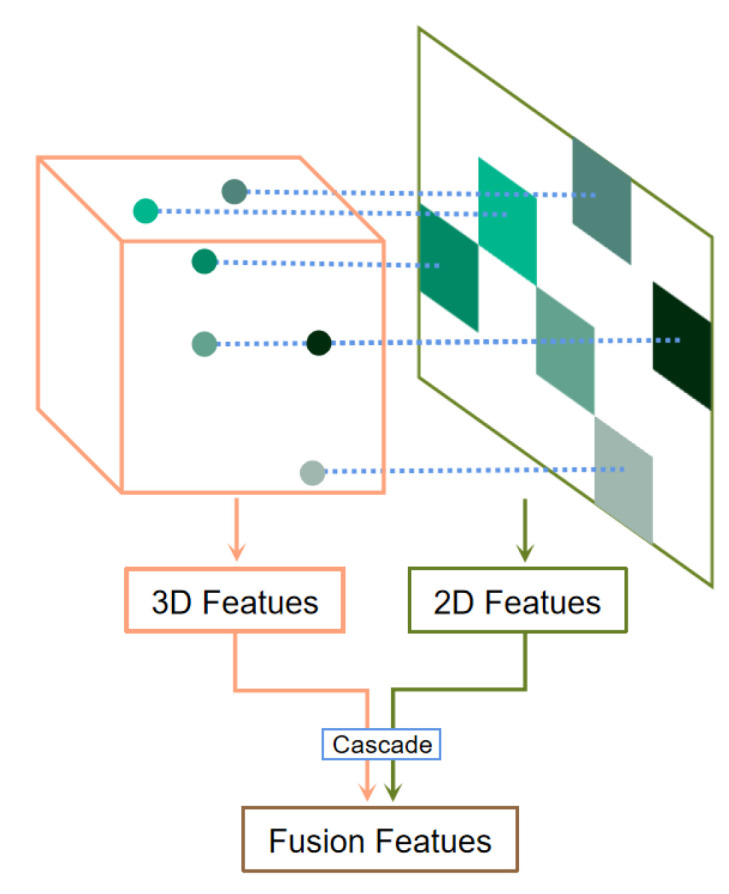
The second fold fusion of SMS.

The feature extraction of the pointcloud within the cube is relatively well understood, but the projected pixels are discontinuous. To solve the problem of being unable to extract the discontinuous pixels, we intercept the input image with the minimum position of rows and columns in the projected pixels as the upper left vertex of the rectangle, and the maximum position of rows and columns as the lower right vertex of the rectangle to obtain a continuous block of pixels. This pixel block corresponds to the pointcloud within the cube, and then feature extraction is implemented to cascade the pointcloud features of the cube with the image features, completing the second fold data fusion in this paper, as schematically shown in Figure 11.

**Figure 12:**
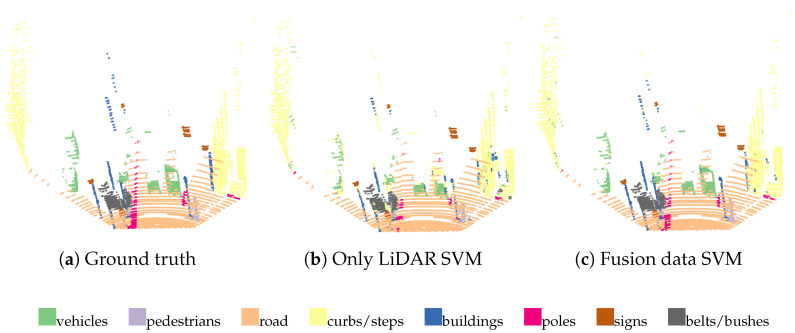
Comparison of classification results based on pointcloud data and fused data from pointcloud images. (**a**) The true value; (**b**) the classification result based on pure pointcloud data; and (**c**) the classification result based on the fusion of pointcloud and image data.

It is assumed that objects in the urban environment can be divided into eight categories, namely vehicles, pedestrians, roads, curbs/steps, buildings, poles, signs, and green belts/bushes. The visualization of the classification results of SVM is given in Figure 12, which compares the classification results using single LiDAR pointcloud data and the classification results using a fusion of LiDAR and camera data.

In the above result visualization, it can be seen that the classification results based on pointcloud data alone are much less effective than those based on fusion data, in which there are some obvious errors. However, the classification results based on fusion data also have some errors, such as cars among walls, the road among cars, the road in the curbs, etc.

**Figure 13:**
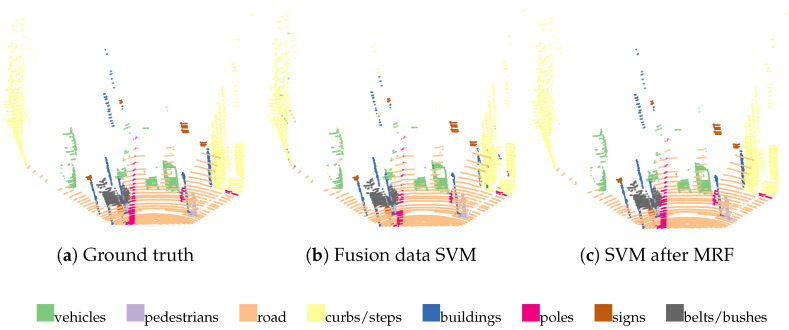
The comparison of classification with MRF model before and after optimization. Where figure (**a**) is the true value, (**b**) is the classification result based on fused data, and (**c**) is the optimized result after fused data classification.

The accuracy of the fusion classification is used as the weight of the svm node on the *C* node in the MRF model, and after optimization, the results are shown in Figure 13. The figure shows that it is obvious that the performance is improved, and those original wrong classifications are corrected into the appropriate categories under the MRF model.

We establish the confusion matrix for the actual and predicted labels, as shown in Table 1:

TP (True Positive) means this class has been predicted as this class successfully, FP (False Positive) means other classes are predicted as this class incorrectly, FN (False Negative) means that this class is predicted as other classes incorrectly, and TN (True Negative) means that other classes are predicted as other classes successfully.

Thus, the following definitions are available:

(18) $$Accuracy=\frac{\mathrm{TP}+\mathrm{TN}}{\mathrm{TP}+\mathrm{TN}+\mathrm{FP}+\mathrm{FN}}$$

(19) $$Precision=\frac{\mathrm{TP}}{\mathrm{TP}+\mathrm{FP}}$$

(20) $$Recall=\frac{\mathrm{TP}}{\mathrm{TP}+\mathrm{FN}}$$

(21) $$F1=\frac{2\times Accuracy\times Recall}{Accuracy+Recall}$$

Table 2 shows the evaluation metrics of several classification results, and the classification results based on fusion data proved to be significantly better than those based on single data. The performance of the fusion results also improved significantly after optimization.

**Figure 14:**
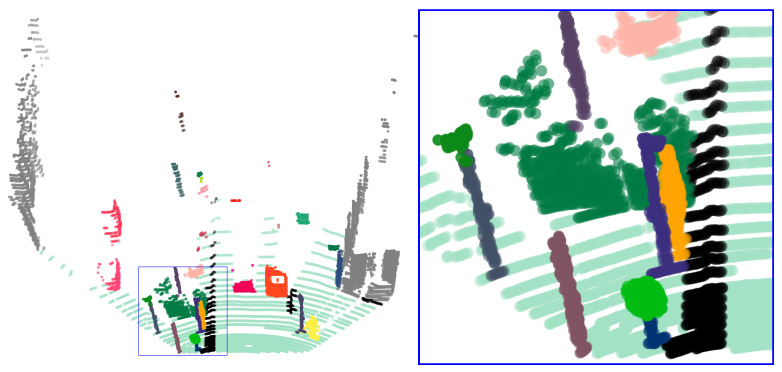
The visualization result of the instance segmentation and the corresponding detail.

Currently, the results are viewed as just a stack of cubes with category labels, which does not actually generate an overall recognition of an object. It is impossible to know in advance how many separated individuals are in the environment, and so it was chosen to conduct instance segmentation with the Mean Shift algorithm. Based on the settings of the categories in this paper, a choice was made to differentiate individuals for vehicles, pedestrians, signs, and poles. Figure 14 shows the visualization result of the instance segmentation and the corresponding detail: according to the original image, it is known that the detailed positions are more varied, with more individuals, and are more crowded, but our segmentation is well-defined.

It should be noted that the reason for not having an instance segmentation ground truth is that our algorithm is only semi-labeled, and this paper has only labeled the categories of the cubes in the fusion space.

## 5. Conclusions

The purpose of this paper is to perform the 3D instance segmentation of urban environments, for which a model named SMS is designed to perform the dual fusion of LiDAR pointcloud data and camera image data, and an MRF model based on spatial contextual relationships is designed to optimize the classification results of the algorithm. It improves the classification accuracy from 94.8% to 99.3%, and the average improvement in all other evaluation data is greater than 5%. Without knowing the number of dissimilar individuals in the environment, our SMS model is able to segment the instances in the urban environment with high accuracy and fast speed. Thus, it is a semi-labeled, lightweight, and efficient 3D instance segmentation model.

Our SMS model has only been tested on the KITTI dataset, and so overfitting or underfitting may occur in some cases; thus, even though SMS does not require precision for point-level labeling when performing instance segmentation, some effort is required for labeling. Moreover, even in a relatively structured urban environment, there are some factors such as bad weather that may cause bad segmentation damage to SMS. Therefore, the robustness of SMS should be further optimized in future work, through approaches such as adding other sensors that are not susceptible to such damage.

## Figures and Tables

**Table 1:** Confusion matrix of actual and predicted labels.

	Predicted		
Actual		Positive	Negative
	**Positive**	TP	FN
	**Negative**	FP	TN

**Table 2:** Comparison of evaluation indicators for different classification results.

	Test Set	The Whole			
	Accuracy	Accuracy	Precision	Recall	F1
Only LiDAR SVM	85.6%	86.9%	87.2%	72.9%	77.6%
Fusion data SVM	95.7%	94.8%	95.3%	90.8%	92.7%
SVM after MRF	×	99.3%	98.6%	98.8%	98.6%

## Data Availability

Not applicable.
